# Estimation of R0 for the spread of SARS-CoV-2 in Germany from excess mortality

**DOI:** 10.1038/s41598-022-22101-7

**Published:** 2022-10-14

**Authors:** Juan Pablo Prada, Luca Estelle Maag, Laura Siegmund, Elena Bencurova, Chunguang Liang, Eleni Koutsilieri, Thomas Dandekar, Carsten Scheller

**Affiliations:** 1 Department of Bioinformatics, Biocenter, Am Hubland, University of Würzburg, 97074 Würzburg, Germany; 2 Institute of Virology and Immunobiology, University of Würzburg, Versbacher Str. 7, 97078 Würzburg, Germany

**Keywords:** SARS-CoV-2, Viral epidemiology

## Abstract

For SARS-CoV-2, R0 calculations in the range of 2–3 dominate the literature, but much higher estimates have also been published. Because capacity for RT-PCR testing increased greatly in the early phase of the Covid-19 pandemic, R0 determinations based on these incidence values are subject to strong bias. We propose to use Covid-19-induced excess mortality to determine R0 regardless of RT-PCR testing capacity. We used data from the Robert Koch Institute (RKI) on the incidence of Covid cases, Covid-related deaths, number of RT-PCR tests performed, and excess mortality calculated from data from the Federal Statistical Office in Germany. We determined R0 using exponential growth estimates with a serial interval of 4.7 days. We used only datasets that were not yet under the influence of policy measures (e.g., lockdowns or school closures). The uncorrected R0 value for the spread of SARS-CoV-2 based on RT-PCR incidence data was 2.56 (95% CI 2.52–2.60) for Covid-19 cases and 2.03 (95% CI 1.96–2.10) for Covid-19-related deaths. However, because the number of RT-PCR tests increased by a growth factor of 1.381 during the same period, these R0 values must be corrected accordingly (R0corrected = R0uncorrected/1.381), yielding 1.86 for Covid-19 cases and 1.47 for Covid-19 deaths. The R0 value based on excess deaths was calculated to be 1.34 (95% CI 1.32–1.37). A sine-function-based adjustment for seasonal effects of 40% corresponds to a maximum value of R0_January_ = 1.68 and a minimum value of R0_July_ = 1.01. Our calculations show an R0 that is much lower than previously thought. This relatively low range of R0 fits very well with the observed seasonal pattern of infection across Europe in 2020 and 2021, including the emergence of more contagious escape variants such as delta or omicron. In general, our study shows that excess mortality can be used as a reliable surrogate to determine the R0 in pandemic situations.

## Introduction

The basic replication number (R0) of a virus describes the average number of secondary infections caused by an infected individual in an immunologically still naive population^1^. R0 is a key factor in predicting the spread of a virus in a population. It is also used to estimate the proportion of individuals required in a population to achieve herd immunity^2^. In addition, the magnitude of R0 can also be used to predict whether a respiratory virus in temperate climates will develop a seasonal pattern of infection (as observed with influenza viruses and endemic coronaviruses) rather than continuous transmission throughout the year^3^.

R0 is influenced not only by intrinsic characteristics of the pathogen, such as its infectivity and mode of transmission, but also by characteristics of the population under study such as the population density^4^. For respiratory viruses, there are several such extrinsic characteristics that have a significant impact on the probability of transmission and thus on R0: The density of a population, the number of persons living in a household and their average vulnerability to infections, other social factors that affect the number of close contacts between infected and uninfected persons (e.g., use of public transportation, work laws when ill, etc.), and also the climate of the area in which the population is located^5^.

Based on data from 425 confirmed cases in Wuhan, R0 of SARS-CoV-2 was estimated to be 2.2^6^. Another report estimating R0 based on case reports in Wuhan yielded a higher R0 of 5.7^7^. This wide range of values is also reflected in a number of other analyses in which R0 was determined to be between 1.95 (WHO estimate) and 6.49 (all reviewed in Ref.^8^). The German Robert-Koch-Institute (RKI) assumes an R0 in the range of 2.8–3.8^9^ based on systematic reviews^10–12^.

All these estimations of R0 have in common that they are based on incidences of SARS-CoV-2 infections detected by RT-PCR. These estimations are therefore not only dependent on the characteristics of the population under study, but also on testing strategies (e.g. representative sampling, symptom-based testing, contact-based testing of index patients, etc.) as well as rapidly increasing numbers of available and performed tests during the early weeks of the pandemic (at least, if no mathematical corrections for this increase were performed).

Because SARS-CoV-2 infections have led to excess mortality in many countries worldwide^13^, the increase in excess mortality can be used as a surrogate for SARS-CoV-2 infections in order to calculate R0 independent of testing strategies and testing capacity. Here, we determined R0 for SARS-CoV-2 infections in Germany during the early phase of the pandemic in February and March 2020 based on Covid-19-associated excess mortality. For comparison, we also calculated R0 from incidence data of SARS-CoV-2 infections corrected by the increase in test capacities, as well as R0 from incidence data of RT-PCR-confirmed Covid-19-related deaths.

## Methods

### Databases

The number of Covid-19 cases, Covid-19-related deaths and SARS-CoV-2-RT-PCR-tests was accessed from the Robert-Koch-Institute (RKI) website^14^. The definition of “Covid-19 case” used here is that of the RKI, which does not use the date of receipt of a positive PCR sample, but rather the date of illness, which in some cases is several days earlier^13^. In accordance with Section 11 (1) of the IfSG (Infektionsschutzgesetz), the public health authorit only reports cases of illness or death and evidence of pathogens that meet the case definition in accordance with Section11 (2) IfSG. Excess mortality was calculated from data of the Federal Statistical Office^15^. All used datasets can be downloaded as excel file from the Supplementary Material S1. Mobility data was taken from the Apple website^16^, which provided the movement data of Apple cell phones from different countries to be used for scientific evaluation in the context of the Covid pandemic. As of April 14, 2022, Apple is no longer providing COVID-19 mobility trends reports. The datafile used for this study can be accessed in the Supplement Sect. S5. All methods were performed in accordance with the Declarations of Helsinki.

### Calculation of excess mortality

To calculate excess mortality per calendar week, the number of weekly deaths in 2020 was subtracted from the mean of weekly deaths in 2016–2019 in line with the definition used by the Federal Statistical Office to calculate excess mortality in Germany^15^. For calculation of “adjusted excess deaths”, the excess mortality in calendar week 10 was tared to 0 in all age groups and the values of the following calendar weeks were adjusted accordingly.

### Calculation of R0

R0 was determined using the R package from Obadia et al.^17^ in R version 3.60. We selected the exponential growth method of the package for calculation of R0. The mean serial interval (average time between successive infection cases) was simulated following a gamma distribution with mean equal to 4.7 (± SD 2.9)^18^. Weekly incidence values of excess mortality were converted to simulated daily incidence values using a gamma distribution. The R-script can be downloaded from the Supplementary Material S2.

## Results

### Determination of the time period that can be used for the calculation of R0

**Figure 1:**
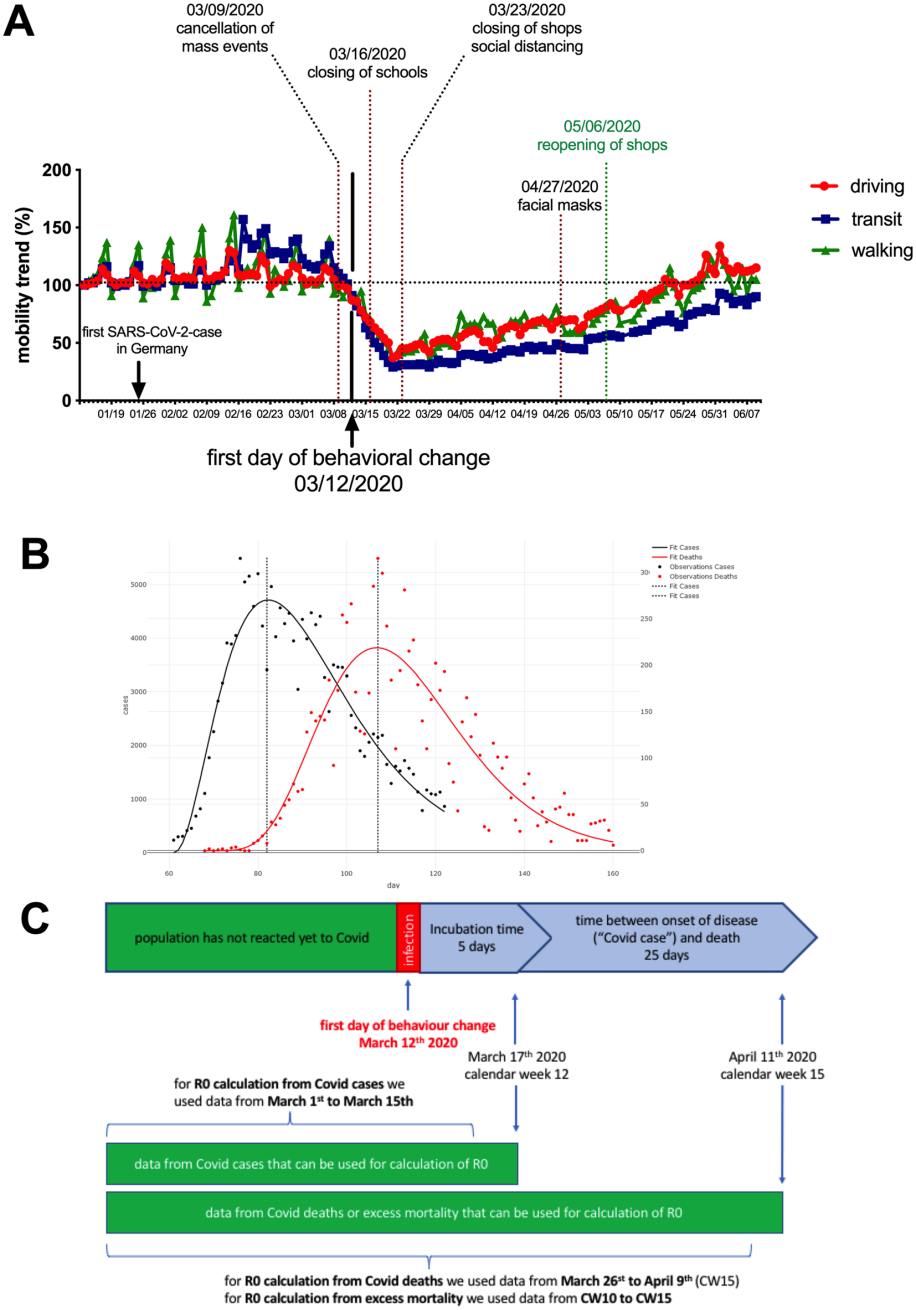
Identifying SARS-CoV-2 datasets unaffected by policies or behavioral changes for estimating R0. (**A**) Mobility data (driving) for Germany, provided by Apple^16^ (driving: red; transit: blue; walking: green). The first change in mobility trends is observed for March 13. (**B**) Data provided by RKI for Covid-cases (black) and Covid-19-related deaths (red) were fitted by gamma distribution. The maxima of the two curves are 25 days apart. (**C**) Graphical representation of the date up to which data from Covid-19 disease cases or Covid-19 death cases can be used to determine R0 without affecting the outcome through policy actions or societal responses.

**Figure 2:**
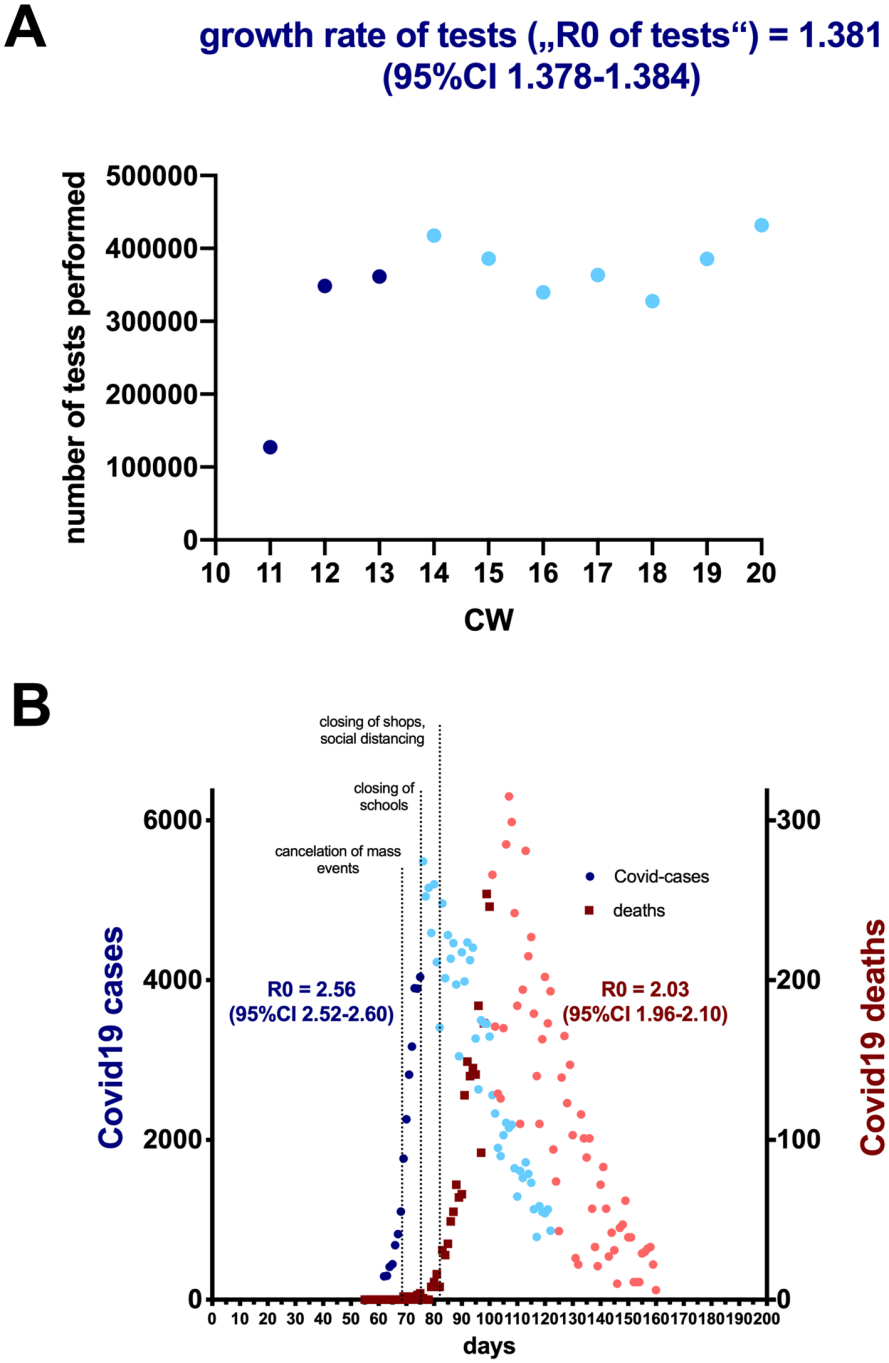
Calculation of R0 from Covid-19 disease incidence numbers and Covid-19 related deaths. (**A**) Data reported by the RKI for the number of performed SARA-CoV-2 RT-PCR-tests. (**B**) Data reported by the RKI for Covid-19-cases (blue symbols, left y-axis) and Covid-19 related deaths (red symbols, right y-axis). (**A,B**) Date were fitted to an exponential growth curve with a serial interval of 4.7 (± SD 2.9) to calculate R0. Dotted lines in B represent the dates for political interventions (03/09/2020 cancellation of mass events, 03/16/2020 closing of schools, 03/23/2020 closing of shops and social distancing). Dark blue and dark red symbols represent data points that were considered for the determination of R0, light blue and light red symbols represent later data points that were not considered for the calculation.

The governments of Germany and its states have taken several measures to contain the SARS-CoV-2 epidemic in Germany in early 2020, including canceling mass events (implemented March 9), closing schools (implemented March 16), closing stores (except grocery stores and pharmacies) and implementing social distancing rules prohibiting personal contact outside the family (implemented March 23) (Fig. 1A). All of these measures, as well as widespread media coverage of the SARS-CoV-2 epidemic in Germany, likely had an impact on the spread of SARS-CoV-2. Therefore, to estimate the value of R0 in Germany, it is imperative to include only data from time points that either predate the implementation of these measures or from time points when these measures could not yet have had an impact on the observed parameter used to calculate R0. As shown in Fig. 1A, people in Germany started to reduce their mobility from March 12, i.e., even a few days earlier than social distancing was officially introduced. Because the incubation period between SARS-CoV-2 infection and the onset of Covid-19 symptoms is on average 5–6 days^9^, behavioral changes can lead to an impact on the number of disease cases no earlier than 5–6 days later (i.e. March 17–18). However, because the number of confirmed SARS-CoV-2 infections already peaked on March 14 (see Fig. 2B) and because we wanted to avoid underestimating R0 in our calculations by possibly including values from an already flattening curve, we added an additional safety margin of 2 days to the first measurable behavioral changes and included for our R0 calculations incidence data of Covid-19 disease cases up to and including March 15 (calendar week 12) without risking that behavioral changes may have had an impact on the R value determined (Fig. 1C).

The RKI provides different epidemiological datasets that can be used for calculations of R0 of SARS-CoV-2 such as daily numbers of detected cases and daily numbers of CoViD-19-related deaths. We fitted the data to a gamma distribution and determined the difference between the peaks of the curves. The mean time between the occurrence of Covid-19 disease cases and Covid-19-related deaths was 25 days (Fig. 1B). Covid-19-related deaths can therefore be used for the determination of R0 at significantly later time points than the occurrence of Covid-19 disease cases without risking of compromising the R0 value by behavioral changes. Therefore, to determine R0 from reported Covid-19 deaths (as well as from Covid-19-related excess mortality), we used records up to and including April 11 (calendar week 15) (Fig. 1C).

### Calculation of R0 from incidence data of Covid-19 disease cases and Covid-19 deaths

In the initial phase of the pandemic, testing capacities were significantly smaller than the actual number of infections. The steep rise in the number of reported cases during this period might therefore also due in significant part to the sharp increase in the number of SARS-CoV-2 RT-PCR test performed. For our calculations of R0, we use incidence data up to and including calendar week 12 for Covid-19 disease cases and up to and including calendar week 15 for Covid-19 deaths. While data for the number of tests performed are not available for the period before calendar week 11, the RKI provides at least the number of tests performed from week 11 onwards^14^. As depicted in Fig. 2A, a significant increase in the number of tests performed can be observed in the calendar weeks 11 to 13. To determine what impact this increase in testing numbers had on reported Covid-19 incidences, we determined the growth rate of testing during this period. The growth rate of testing yields an “R0 of tests” of 1.38 (Fig. 2A), meaning that even if the number of infections remained constant during this period of time, an apparent increase of 1.38 in R0 would be observed. It follows that R0 values from incidence figures must be corrected by this factor.

From the raw incidence data, we obtain an R0 of 2.56 for Covid-19 disease cases and an R0 of 2.03 for Covid-19 death cases (Fig. 2B). However, these values must still be corrected for the growth rate of testing (R0_corrected_ = R0_uncorrected_/“R0 of tests”), resulting in a corrected R0 of 1.86 for Covid-19 disease cases and an R0 of 1.47 for Covid-19-death cases. The R0 value derived from deaths is slightly lower than the R0 value determined from Covid-19 incidence values. This may be due to the fact that in the initial phase of the pandemic, severely ill cases (and thus individuals at higher risk of death) were preferentially tested, while milder and asymptomatic cases were increasingly included in testing as the testing capacity expanded. Such a change in testing strategy inevitably introduces a bias toward higher R0 values when calculated from Covid-19 incidence data compared with Covid-19 death data.

However, even if we correct the incidence values for test capacity dynamics, this way of determining R0 still remains subject to many uncertainties: First, the exact numbers of tests performed in the first weeks of the pandemic were not collected for Germany, so that an accurate estimate of the dynamics of testing capacity is not possible. Second, the incidence data do not come from representative samples in the general population, but mainly from symptomatic patients and persons with whom they came into contact. Therefore, this dataset contains a disproportionate number of infections from nursing homes and hospitals, where symptomatic infections are overrepresented and where transmission probabilities are most likely different from what would be expected in the general population. Therefore, the R0 values calculated above are unlikely to be representative of the spread of the virus in the general population.

### Calculation of R0 from excess mortality

To address this problem, we also determined R0 based on excess mortality data in Germany during early 2020. The Federal Statistical Office of Germany lists all deaths that occur in Germany, regardless of their cause^15^. Because SARS-CoV-2 infection has led to increased excess mortality in many countries, these data can be used as surrogate markers for the spread of SARS-CoV-2 infections. And because excess mortality is independent of the number or strategy of SARS-CoV-2 testing, it provides a representative picture for the spread of infections in the general population.

**Figure 3:**
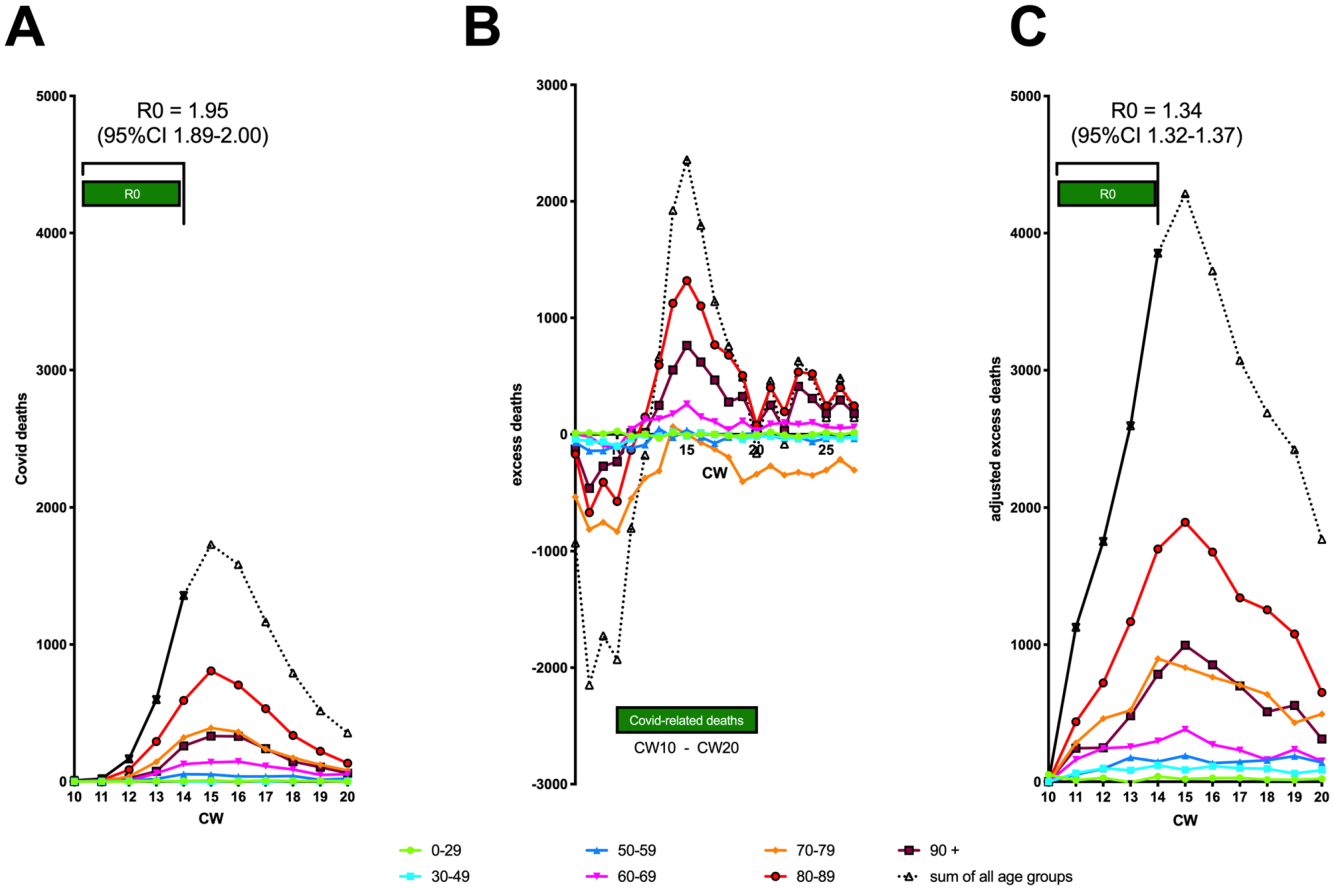
Calculation of R0 from excess mortality. (**A**) Covid-19 related deaths as weekly incidence in different age groups. (**B**) Excess deaths in 2020 in different age groups based on comparison with average weekly deaths in 2016–2019. (**C**) Excess deaths presented in B, but adjusted to 0 for week 10 in each age group, so that relative changes related to Covid-19 become better visible.

Figure 3A shows the incidence of deaths with confirmed SARS-CoV-2 infection. The data set shown here is the same as that in Fig. 2B, but this time as weekly incidence and subdivided into different age groups. The (uncorrected) R0 value here is 1.95, similar to 2.03 from Fig. 2B. From this figure, it can be seen that the peak of Covid-19 related mortality is between calendar week 10 and 20. Figure 3B shows excess mortality (in relation to average weekly deaths in 2016–2019) in the different age groups, and one can see a parallel trend to the confirmed Covid-related deaths between calendar weeks 10 and 20. Based on the respective values of calendar week 10, from which an increase in excess mortality is observed in all Covid-19 relevant age groups, we plotted the change in all values in Fig. 3C. From this adjusted excess mortality, we obtained an R0 of 1.34 (95% CI 1.32–1.37) for the sum of all age groups for the spread of SARS-CoV-2 in the general population in Germany. For the individual age groups, the R0 values were very similar: age 90+: 1.38 (1.34–1.42), age 80–89: 1.37 (1.35–1.40), age 70–79: 1.31 (1.26–1.36), age 60–69: 1.22 (1.16–1.29), age 50–59: 1.59 (1.45–1,74), age 30–49: 1.16 (1.06–1.26), age 0–29: 1.89 (95% CI 047–5.56).

### Influence of influenza-related excess deaths on Covid-19 related excess deaths

**Figure 4:**
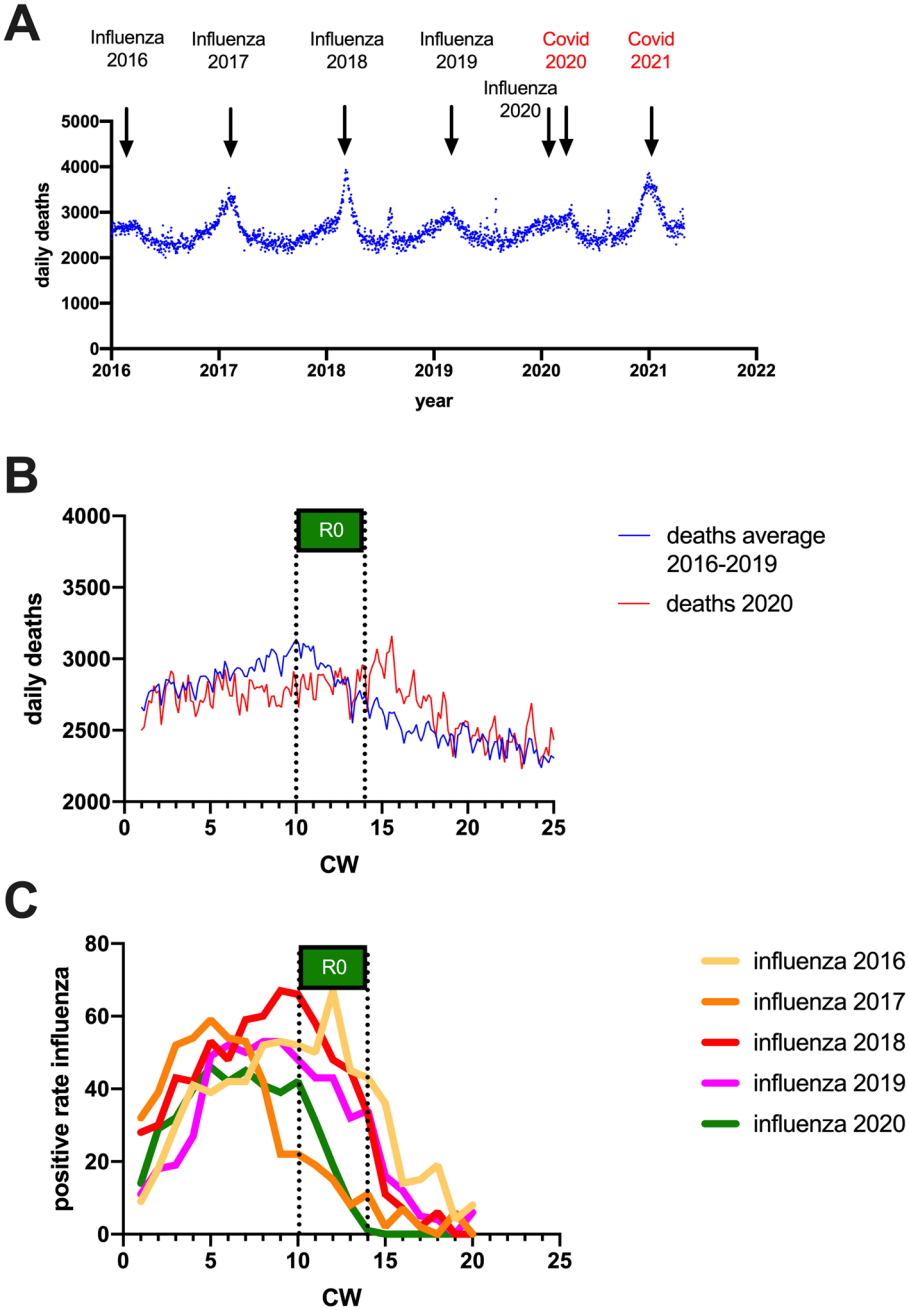
Comparison of Influenza-related and Covid-19 related mortality in Germany. (**A**) Daily deaths in Germany from 2016 to 2021 (data taken from the Federal Statistical Office of Germany). (**B**) Comparison of daily number of deaths in 2020 (red line) with average number of daily deaths in 2016–2019 (blue line). (**C**) Positive rate for influenza infections in Germany for calendar weeks 1–20 from 2016 to 2020 (data taken from the RKI influenza survey).

Due to the lack of representative measurements, Covid-19 related excess mortality is the only infection parameter that is free from bias due to changes in testing strategy or testing numbers. However, excess mortality data are subject to other confounding factors that may have an impact on the calculation of R0: The Covid-19 pandemic reached Germany at a time when seasonal influenza activity in Germany was already subsiding (see Fig. 4C). Influenza-related excess mortality and Covid-19 related excess mortality are therefore superimposed in the total excess mortality data sets. If influenza mortality had been significantly elevated in 2020 compared with previous years (2016–2019), this could mask Covid-19 related effects, particularly if influenza mortality rates that were already falling again coincided with an incipient increase in Covid-19 mortality rates. However, a look at mortality in previous years shows that influenza-related mortality in Germany in 2020 was significantly lower compared with previous years because of the two exceptionally strong influenza years 2017 and 2018 (Fig. 4A). As a result, under-mortality was observed in Germany in calendar weeks 10–14 compared with previous years, rather than excess-mortality (Fig. 4B). Thus, if there was an effect of influenza-related deaths on the calculation of R0 for Covid-19 infections, it was one that resulted in an overestimate of R0 rather than an underestimate. Thus, the R0 calculated here of 1.34 (95% CI 1.32–1.37) should be regarded as a maximum value, whereas the actual R0 of SARS-CoV-2 infections in Germany is likely to be even lower.

### Seasonal effects on R0

**Figure 5:**
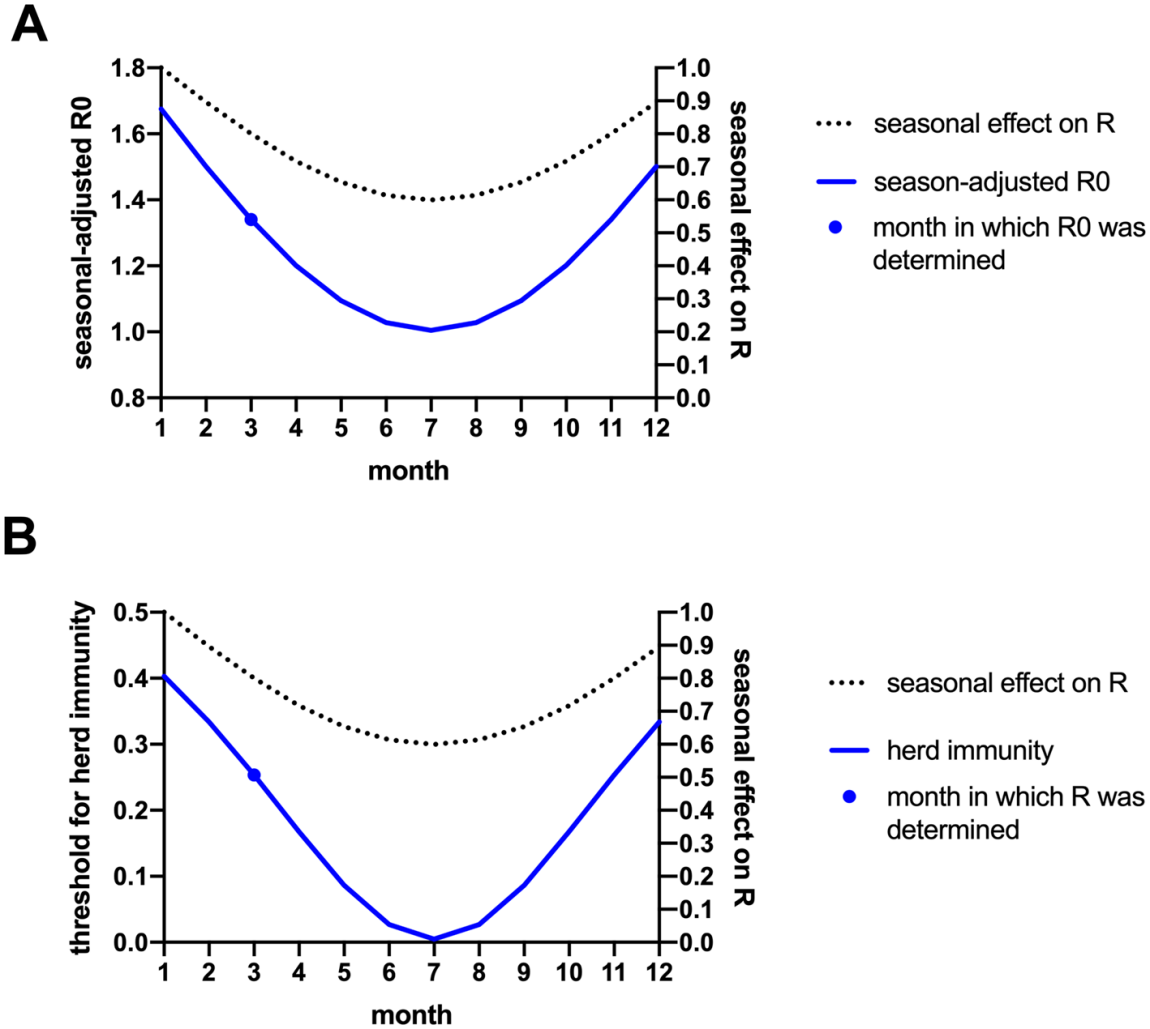
Seasonal influence on R0 and herd immunity. (**A**) The seasonal effect on R0 can be assumed as a sine function with a maximum in January and a 40% lower minimum in July (dashed line, right y-axis). This translates into an oscillating R0 with a maximum in January (of R0_January_ = 1.68) and a minimum in July (R0_July_ = 1.01) (blue line, left y-axis). R0 calculations are based on the values calculated for March (R0_March_ = 1.34) (blue dot) (**B**) Herd immunity similarly to R0 oscillates in a seasonal pattern.

The seasonal effect on R0 can be approximated as a sinusoidal pattern with a maximum in January as the coldest month in Germany and a minimum in July as the warmest month, with an approximate 40% reduction in July compared to January^3^ (Fig. 5, dotted line). As our calculation of R0 was based on the infection situation in March 2020, it can be expected that the R0 value determined in this way is about 20% lower than the maximum value that would be reached in January if the pandemic would have reached Germany earlier. According to this model, R0 determined in March with a value of R0_March_ = 1.34 would reach its maximum in January with a value of R0_January_ = 1.68 and fall to a minimum of R0_July_ = 1.01 in July (Fig. 5A).

Herd immunity is dependent on R0 (herd immunity = (1 − 1/R0)), and therefore also fluctuates seasonally. Thus, herd immunity against the original SARS-CoV-2 strain would oscillate between 40% in January and below 1% in July (Fig. 5B). This explains why the number of SARS-CoV-2 infections in summer 2020 not only declined again in “lockdown” countries such as Germany (and remained low in these countries even after the suspension of policy measures during the summer), but also why the same seasonal pattern was observed in countries with little or no countermeasures against SARS-CoV-2^13^.

With a seasonal increase in the threshold of herd immunity, the following winter (2020/2021) fueled again the spread of SARS-CoV-2 Germany, until infection numbers dropped again in spring 2021. At this time, about 3.8 million SARS-CoV-2 infections have been reported to the RKI, corresponding to 4.5% of the population (May 2021)^19^ and a serological survey of blood donors revealed a seropositivity rate of 14% in April 2021^20^, showing a substantial underestimation of SARS-CoV-2 infections from RT-PCR-data alone. Together with the enrollment of the Covid-vaccine campaign, immunity in the German population has reached up to 40% by end of May 2021, and that coincided with the emergence of the delta variant, which can now be interpreted as an escape variant that overcame the 40% herd immunity restrictions of the original SARS-CoV-2 strains by higher contagiousness (and therefore also a higher R0).

Since the population in Germany was no longer naïve towards SARS-CoV-2 during summer of 2021 when the delta variant began its expansion in Germany, it is not possible to determine an R0 for this variant. However, the RKI calculates daily R_e_ values based on a 7-day period for Germany, and the values for the delta variant reached 1.3 during July/August 2021^21^. With a seasonal variation of 40%, this value corresponds to a theoretic maximum in December with an R_e_ = 2.2, translating into a 55% threshold for winter herd immunity.

## Discussion

The early SARS-CoV-2 infection spread in Germany with an R0 of 1.34 (95% CI 1.32–1.37). This value is much lower than what had been expected based on R0 determinations from the literature, where values between 2 and 3 became consensus^8,9^. Although the German RKI has not published an R0 estimation for Germany, it provided daily estimations of R based on a four-day-period. These daily R values during the first two weeks of March 2020 were in the range of 2.2–3.2^22^. Based on the reporting data for positive RT-PCR results from the “our world in data” database of Oxford University, an R0 of 3.37 was determined for Germany^23^, but these calculations used RT-PCR reporting data rather than data for Covid-19 disease cases and therefore are not fully comparable with the calculations from the RKI. The discrepancies between these high values and the rather low R0 estimates in our manuscript are primarily due to the fact that in these earlier estimations the R0 values were not corrected by a factor accounting for the substantial increase in test capacity during this period. If we use our uncorrected R0 estimate based on Covid-19 case numbers for comparison (R0 = 2.56, Fig. 2B), it is in the same order of magnitude as the values calculated by the RKI.

A high R0 of the order of 3 would likely have resulted in a lack of seasonal progression, as a seasonal effect was estimated to reduce R0 by only 40% based on observations in endemic coronaviruses^3^. Accordingly, health authorities expected an unrestrained spread of the virus for Germany, whereupon a series of policy measures were adopted aiming to actively reduce the incidence of infection. In retrospect, however, a clearly seasonal occurrence is evident not only for Germany, but also for all other countries in temperate climates, in particular also for Sweden, where hardly any measures have been taken to contain the spread of SARS-CoV-2 in the general population^13^.

The core of this study is the calculation of R0 from excess mortality data. Although this type of calculation is free of sources of bias that affect conventional calculations of R0 from infection incidence data (e.g., increase in number of tests, changes in testing strategy, increase in reporting awareness), we would like to point out some limitations in the presenting study as well: Excess mortality is dependent on multiple factors, and pandemic-related medical shortages could lead to an increase in excess mortality that is independent of a direct effect of SARS-CoV-2. Such a bias would lead to an overestimation of R0. However, we believe that such an effect, if present at all, is likely to have played only a minor role: First, there was no significant reduction in medical care in Germany during the Covid pandemic, so no significant secondary effects on mortality rates would be expected. Moreover, our R0 calculations are significantly lower than most other calculations, so that empirically such an effect is unlikely to have played a major role. Another point is that the calculation of excess mortalities takes into account not only the striving figures of the respective period under consideration (in our case spring 2020), but also the mortality figures of a reference period before that (in our case the past 4 years 2016–2019). An increase or decrease in excess mortality in the period under consideration is therefore always dependent on the development of death rates in previous years. We have tried to consider this bias at least qualitatively (see Fig. 4), showing that this effect might have led to an overestimation of R0 in our calculation. The use of excess mortality as a surrogate for the spread of infection in a population requires the assumption that the proportion of particularly vulnerable groups (the elderly and patients with preexisting conditions) in the total incidence of infection does not change significantly during the analysis period. Data from the RKI on the age distribution of Covid infections show a constant age distribution of infections in the early phase (weeks 10 to 12) of the pandemic in Germany^24^, so we can assume that the number of Covid-related deaths is indeed a reliable surrogate marker for infection incidence. Factors such as changing seroprevalence or changing variants of SARS-CoV-2 were not included in the calculation of R0. This simplification seems appropriate to us because in the early phase of the pandemic, seroprevalence was still very low and thus could have only marginal influence on the spread of infection. In addition, at the time of analysis (until week 12 in 2020), in Germany and Europe there were almost exclusively the very closely related SARS-CoV-2 clades 20A, 20B, 20C and 20D, for which a similar transmissibility can be assumed^25^. Finally, the calculation of R0 depends on the size of the serial interval: the larger the serial interval, the larger the calculated value for R0. A large number of articles have now appeared in the literature determining the serial interval of SARS-CoV-2 infection at begin of the pandemic. In a meta-analysis of 56 articles, a range of 1–9.99 was determined^18^. The German RKI assumes a serial interval of 4 days in its estimates for calculating Re. The SI of 4.7 days chosen in this paper thus leads to a slight overestimation of R0 compared to the parameters used by the RKI. In Supplement 4 we have shown a calculation of R0 for different SI.

The concept of herd immunity in respiratory pathogens such as coronaviruses does not imply permanent protection of the population against seasonal reemergence of these pathogens, since the immunity achieved may decrease over time, especially in asymptomatically infected patients^26^. Instead, the achievement of herd immunity in respiratory viruses leads to a strong selection pressure for escape mutations (classical immune escape or increased contagiousness), which can then give rise to new waves of infection^27^. For this reason, respiratory viruses such as influenza- or coronaviruses remain endemic, despite broad immunity, which will probably also be the case for SARS-CoV-2.

## Conclusion

Our study shows that the R0 value of SARS-CoV-2 can be calculated from excess mortality data. We also introduce here the concept of a seasonally adjusted R0 value, which should be reported as a range (R0_January_–R0_July_) rather than a static value. We determined an R0 value of 1.34 for infections in March 2020 (R0_March_ = 1.34), corresponding to a seasonal range of R0_January_ = 1.68 and a minimum in July (R0_July_ = 1.01). This rather low range of R0 values is much more consistent with observations of pandemic progression than many earlier and much higher estimates of the R0 value. The massive expansion of testing capacity in the early phase of the pandemic, combined with changes in testing strategy, was a major cause of the overestimation of the R0 value. Excess mortality can be determined independently of SARS-CoV-2 testing capacity in many countries, and therefore can be a valuable tool in future pandemics to provide reliable values for the rate of spread of an emerging pathogen in a population when representative samples of pathogen spread are not available.

## Supplementary Information

Supplementary Information 1.

Supplementary Information 2.

Supplementary Information 3.

Supplementary Information 4.

Supplementary Information 5.

## Data Availability

All data generated or analysed during this study are included in this published article and its Supplementary Information files.
