# Supplementary material for: Estimation of R0 for the spread of SARS-CoV-2 in Germany from excess mortality
Source: Sci Rep. 2022 Oct 14;12:17221. doi: 10.1038/s41598-022-22101-7 (PMC9562071; doi:10.1038/s41598-022-22101-7)
Supplement: Supplementary file 2 — Supplementary Information 2. [file 41598_2022_22101_MOESM2_ESM.docx]

## script that accompany the supplemental material of the paper Estimation of R0 for the spread of SARS-CoV-2 in Germany from Excess Mortality

## by Prada et. al.

## load the required packages, if not present these must be installed #########

require(readxl)

require(R0)

##### replace here the path where the file datasheet_R0 is #######################

path = "D:/Project-covid19/datasheet_R0.xlsx"

######################################### tests performed Fig. 2A #############################################################

test_performed_per_week <- read_excel(path,sheet = "tests performed Fig. 2A")[1:3,1:2]

##### re-structure the data to daily incidence

daily_incidences = matrix(0,ncol = ncol(test_performed_per_week),nrow = nrow(test_performed_per_week)*7)

pos_rows = seq(1,nrow(test_performed_per_week)*7,7)

for (ic in 1:ncol(test_performed_per_week)) {

for (ir in 1:nrow(test_performed_per_week)) {

daily_incidences[pos_rows[ir]:(pos_rows[ir]+6),ic] = rmultinom(1, size = as.numeric(test_performed_per_week[ir,ic]), prob = c(1/7,1/7,1/7,1/7,1/7,1/7,1/7))

}

}

colnames(daily_incidences) = c("day","number of tests")

daily_incidences[,1] = 1:(nrow(test_performed_per_week)*7)

##### calculation of the RO value

GT.flu_w <- generation.time("gamma", c(4.7,2.9)) # daily time

R0 <- estimate.R(daily_incidences[,2], GT=GT.flu_w, methods=c("EG"))

print(paste("Tests performed Fig. 2A estimated R0 = ",R0$estimates$EG$R, " CI (",R0$estimates$EG$conf.int[1]," - ",R0$estimates$EG$conf.int[2],")",sep=""))

######################################### Covid-cases and deaths Fig. 2B #############################################################

covid_deaths_daily <- read_excel(path,sheet = "Covid-cases and deaths Fig. 2B")[1:46,1:2]

covid_cases_daily <- read_excel(path,sheet = "Covid-cases and deaths Fig. 2B")[8:21,c(1,3)]

##### calculation of the RO value

GT.flu_d <- generation.time("gamma", c(4.7,2.9)) # daily time

R0_d <- estimate.R(covid_deaths_daily$`covid deaths`, GT=GT.flu_d, methods=c("EG"))

print(paste("Covid-deaths Fig. 2B estimated R0 = ",R0_d$estimates$EG$R, " CI (",R0_d$estimates$EG$conf.int[1]," - ",R0_d$estimates$EG$conf.int[2],")",sep=""))

R0_c <- estimate.R(covid_cases_daily$`covid cases`, GT=GT.flu_d, methods=c("EG"))

print(paste("Covid-cases Fig. 2B estimated R0 = ",R0_c$estimates$EG$R, " CI (",R0_c$estimates$EG$conf.int[1]," - ",R0_c$estimates$EG$conf.int[2],")",sep=""))

######################################### Covid-deaths Fig. 3A #############################################################

ExcDeaths_per_week <- read_excel(path,sheet = "Covid-deaths Fig. 3A")[1:4,1:9]

##### re-structure the data to daily incidence

daily_incidences = matrix(0,ncol = ncol(ExcDeaths_per_week),nrow = nrow(ExcDeaths_per_week)*7)

pos_rows = seq(1,nrow(ExcDeaths_per_week)*7,7)

for (ic in 1:ncol(ExcDeaths_per_week)) {

for (ir in 1:nrow(ExcDeaths_per_week)) {

daily_incidences[pos_rows[ir]:(pos_rows[ir]+6),ic] = rmultinom(1, size = as.numeric(ExcDeaths_per_week[ir,ic]), prob = c(1/7,1/7,1/7,1/7,1/7,1/7,1/7))

}

}

colnames(daily_incidences) = colnames(ExcDeaths_per_week)

daily_incidences[,1] = 1:(nrow(ExcDeaths_per_week)*7)

##### calculation of the RO value

GT.flu_d <- generation.time("gamma", c(4.7,2.9)) # daily time

print("Excess deaths Fig. 3A results")

for (ind_col in 2:ncol(daily_incidences)) {

final = floor(length(daily_incidences[,ind_col]))

R0 <- estimate.R(daily_incidences[,ind_col], GT=GT.flu_d, methods=c("EG"),begin = 1,end=final)

print(paste(colnames(daily_incidences)[ind_col]," excess deaths estimated R0 = ",R0$estimates$EG$R, " CI (",R0$estimates$EG$conf.int[1]," - ",R0$estimates$EG$conf.int[2],")",sep=""))

}

######################################### Adjusted Excess deaths Fig. 3C #############################################################

AdExcDeaths_per_week <- read_excel(path,sheet = "Adjusted Excess deaths Fig. 3C")[1:4,1:9]

AdExcDeaths_per_week[AdExcDeaths_per_week<0] = 0 # excess death can be bellow 0 but the incidence cannot

##### re-structure the data to daily incidence

daily_incidences = matrix(0,ncol = ncol(AdExcDeaths_per_week),nrow = nrow(AdExcDeaths_per_week)*7)

pos_rows = seq(1,nrow(AdExcDeaths_per_week)*7,7)

for (ic in 1:ncol(AdExcDeaths_per_week)) {

for (ir in 1:nrow(AdExcDeaths_per_week)) {

daily_incidences[pos_rows[ir]:(pos_rows[ir]+6),ic] = rmultinom(1, size = as.numeric(AdExcDeaths_per_week[ir,ic]), prob = c(1/7,1/7,1/7,1/7,1/7,1/7,1/7))

}

}

colnames(daily_incidences) = colnames(AdExcDeaths_per_week)

daily_incidences[,1] = 1:(nrow(AdExcDeaths_per_week)*7)

##### calculation of the RO value

GT.flu_d <- generation.time("gamma", c(4.7,2.9)) # daily time

print("Adjusted excess deaths Fig. 3B results")

for (ind_col in 2:ncol(daily_incidences)) {

R0 <- estimate.R(daily_incidences[,ind_col], GT=GT.flu_d, methods=c("EG"))

print(paste(colnames(daily_incidences)[ind_col]," adjusted excess deaths estimated R0 = ",R0$estimates$EG$R, " CI (",R0$estimates$EG$conf.int[1]," - ",R0$estimates$EG$conf.int[2],")",sep=""))

}
