# Supplementary material for: Estimation of R0 for the spread of SARS-CoV-2 in Germany from excess mortality
Source: Sci Rep. 2022 Oct 14;12:17221. doi: 10.1038/s41598-022-22101-7 (PMC9562071; doi:10.1038/s41598-022-22101-7)
Supplement: Supplementary file 3 — Supplementary Information 3. [file 41598_2022_22101_MOESM3_ESM.docx]

The script attached should simply be run on R, please simply adjust the path to the “datasheet_R0.xlsx” file.

This is the R session info:

**R version 3.6.0 (2019-04-26)**

**Platform: x86_64-w64-mingw32/x64 (64-bit)**

**Running under: Windows >= 8 x64 (build 9200)**

**Matrix products: default**

**locale:**

**[1] LC_COLLATE=German_Germany.1252 LC_CTYPE=German_Germany.1252 LC_MONETARY=German_Germany.1252 LC_NUMERIC=C LC_TIME=German_Germany.1252**

**attached base packages:**

**[1] stats graphics grDevices utils datasets methods base**

**other attached packages:**

**[1] R0_1.2-6 MASS_7.3-53.1 readxl_1.3.1**

**loaded via a namespace (and not attached):**

**[1] Rcpp_1.0.6 fansi_0.4.2 utf8_1.2.1 crayon_1.4.1 cellranger_1.1.0 lifecycle_1.0.0 magrittr_2.0.1 pillar_1.6.0 cli_2.5.0**

**[10] rlang_0.4.10 rstudioapi_0.13 vctrs_0.3.7 ellipsis_0.3.1 tools_3.6.0 yaml_2.2.1 compiler_3.6.0 pkgconfig_2.0.3 tibble_3.1.1**
