# Supplementary material for: Estimation of R0 for the spread of SARS-CoV-2 in Germany from excess mortality
Source: Sci Rep. 2022 Oct 14;12:17221. doi: 10.1038/s41598-022-22101-7 (PMC9562071; doi:10.1038/s41598-022-22101-7)
Supplement: Supplementary file 4 — Supplementary Information 4. [file 41598_2022_22101_MOESM4_ESM.docx]

S3

**The influence of the serial interval on R0.**

| **serial interval in days (± S.D.)** | **R0 (95% CI)** |
| --- | --- |
| 1 (±0.61) | 1.108 (1.103-1.113) |
| 2 (±1.23) | 1.158 (1.150-1.165) |
| 3 (±1.85) | 1.224 (1.214-1.235) |
| 4 (±2.47) | 1.291 (1.277-1.305) |
| 4.7 (±2.90)^1^ | 1.339 (1.132-1.356) |
| 5 (±3.09) | 1.360 (1.343-1.356) |
| 6 (±3.70) | 1.432 (1.411-1.454) |
| 7 (±4.32) | 1.507 (1.481-1.533) |
| 8 (±4.94) | 1.584 (1.554-1.614) |
| 9 (±5.55) | 1.663 (1.629-1.698) |
| 10 (±6.17) | 1.745 (1.706-1.785) |

The length of the serial interval (SI; mean interval between the onset of disease of an infectious case to the onset of disease of a case infected by it) influences the calculated value of R0. Smaller values for SI result in smaller R0 values.

In this manuscript, we used a serial interval for the spread of SARS-CoV-2 of 4.7 days (± 2.9) as estimated from Nishiura et al.^1^. The RKI uses a serial interval of 4 days in its calculations for estimating Re in Germany^2^. Compared with the parameters of the RKI, the R0 in our work is thus slightly overestimated (with an SI of 4 days, our calculations based on excess mortality data would yield an R0 of 1.29, compared with the R0 of 1.34 calculated in the manuscript with an SI of 4.7 days).

In a large meta-review by Ali et al.^3^, a range of SI was derived from 56 articles from 1 to 9.99. Therefore, to illustrate the influence of the selected SI on the calculation of R0, we calculated R0 from the excess mortality data for different SI values between 1 and 10 (see Table S3). The standard deviation of the serial intervals for values 1-10 was adjusted from the standard deviation for the value 4.7: SD_x_ = 2.9 * SI_x_/4.7.

1. Nishiura H, Linton NM, Akhmetzhanov AR. Serial interval of novel coronavirus (COVID-19) infections. *Int J Infect Dis*. 2020;93:284-286. doi:10.1016/j.ijid.2020.02.060
2. Rki. 17 Epidemiologisches 2020 Bulletin. 2020;(April).
3. 3. Nishiura H, Linton NM, Akhmetzhanov AR. Serial interval of novel coronavirus (COVID-19) infections. *Int J Infect Dis*. 2020;93:284-286. doi:10.1016/j.ijid.2020.02.060
